# The Sclerophyllous *Eucalyptus camaldulensis* and Herbaceous *Nicotiana tabacum* Have Different Mechanisms to Maintain High Rates of Photosynthesis

**DOI:** 10.3389/fpls.2016.01769

**Published:** 2016-11-24

**Authors:** Wei Huang, You-Gui Tong, Guo-Yun Yu, Wei-Xian Yang

**Affiliations:** ^1^Key Laboratory of Tropical Forest Ecology, Xishuangbanna Tropical Botanical Garden, Chinese Academy of SciencesMengla, China; ^2^Department of Economic Plants and Biotechnology, Kunming Institute of Botany, Chinese Academy of SciencesKunming, China; ^3^Forestry Bureau of Dongchuan CountyKunming, China

**Keywords:** alternative electron flow, CO_2_ assimilation, mesophyll conductance, photorespiration, sclerophyllous

## Abstract

It is believed that high levels of mesophyll conductance (*g*_m_) largely contribute to the high rates of photosynthesis in herbaceous C_3_ plants. However, some sclerophyllous C_3_ plants that display low levels of *g*_m_ have high rates of photosynthesis, and the underlying mechanisms responsible for high photosynthetic rates in sclerophyllous C_3_ plants are unclear. In the present study, we examined photosynthetic characteristics in two high-photosynthesis plants (the sclerophyllous *Eucalyptus camaldulensis* and the herbaceous *Nicotiana tabacum*) using measurements of gas exchange and chlorophyll fluorescence. Under saturating light intensities, both species had similar rates of CO_2_ assimilation at 400 μmol mol^−1^ CO_2_ (*A*_400_). However, *E. camaldulensis* exhibited significantly lower *g*_m_ and chloroplast CO_2_ concentration (*C*_c_) than *N. tabacum*. A quantitative analysis revealed that, in *E. camaldulensis*, the *g*_m_ limitation was the most constraining factor for photosynthesis. By comparison, in *N. tabacum*, the biochemical limitation was the strongest, followed by *g*_m_ and *g*_s_ limitations. In conjunction with a lower *C*_c_, *E. camaldulensis* up-regulated the capacities of photorespiratory pathway and alternative electron flow. Furthermore, the rate of alternative electron flow was positively correlated with the rates of photorespiration and ATP supply from other flexible mechanisms, suggesting the important roles of photorespiratory pathway, and alternative electron flow in sustaining high rate of photosynthesis in *E. camaldulensis*. These results highlight the different mechanisms used to maintain high rates of photosynthesis in the sclerophyllous *E. camaldulensis* and the herbaceous *N. tabacum*.

## Introduction

In C_3_ plants, rates of photosynthesis differ widely among species. For individual leaves or whole plants, photosynthetic capacity mainly depends upon their biochemical composition and morphology. Generally, plants with high rates of CO_2_ assimilation have higher levels of cytochrome *f*, ATP synthase, Rubisco, and other Calvin-Benson cycle enzymes (Evans, 1987; Terashima and Evans, 1988; Hikosaka, 1996; Hikosaka and Terashima, 1996; Yamori et al., 2010, 2011). The rate of CO_2_ assimilation is maximized in leaves that usually have high levels of stomatal conductance (*g*_s_) and mesophyll conductance (*g*_m_), which increase CO_2_ diffusion into the chloroplasts (Yamori et al., 2010, 2011). Herbaceous plants, e.g., tobacco (*Nicotiana tabacum*), spinach (*Spinacia oleracea*), rice (*Oryza sativa*), and *Triticum aestivum* have commonly been used for studying the mechanism of photosynthetic acclimation and the rate-limiting step for CO_2_ assimilation. In herbaceous C_3_ plants, the rate-limiting step of photosynthesis depends on leaf N content and is mainly determined by N portioning between Rubisco and photosynthetic electron transport (Yamori et al., 2011). However, little is known about the coordination of photosynthetic electron flow and gas exchange in sclerophyllous plants that have high rates of photosynthesis. What is more, it is still not well understood whether the mechanisms responsible for high rates of photosynthesis vary among sclerophyllous and herbaceous C_3_ plants.

Leaf anatomy plays an important role in determining photosynthetic capacity. The area of chloroplasts facing the intercellular space largely determines the light-saturated rate of photosynthesis (Oguchi et al., 2003, 2005). High-photosynthesis herbaceous plants usually have thinner cell walls, leading to high values of *g*_m_. By comparison, leaves of sclerophyllous plants have thicker cell walls, with a high leaf dry mass per area (LMA) (Hassiotou et al., 2009). As an important morphological trait, LMA is inversely related to *g*_m_ in sclerophyllous plants (Hassiotou et al., 2009). Therefore, sclerophyllous plants usually have low *g*_m_ and slow rates of photosynthesis (Loreto et al., 1992). For example, a sclerophyllous species *Quercus guyavifolia* has a low rate of the maximum photosynthesis being 13 μmol CO_2_ m^−2^ s^−1^ (Huang et al., 2016). As we known, the sclerophyllous species *Eucalyptus camaldulensis* has been introduced for production of paper in China due to its high rate of photosynthesis. The high value of LMA for leaves of *E. camaldulensis* is assumed to result in low *g*_m_. Mesophyll conductance plays an important role in determining the rate of photosynthesis in C_3_ plants (Flexas et al., 2008; Carriqui et al., 2015). A low *g*_m_ value increases the resistance of CO_2_ conductance to the chloroplasts, leading to a decline in the chloroplast CO_2_ concentration (*C*_c_) and, thus, restricted CO_2_ assimilation (Loreto et al., 1992; Hanba et al., 2002; Flexas et al., 2012; Gago et al., 2013; Carriqui et al., 2015). Therefore, for the sclerophyllous *E. camaldulensis*, other mechanisms favoring CO_2_ diffusion must be used to increase *C*_c_ because it is essential for the maintenance of high CO_2_ assimilation.

The net rate of CO_2_ assimilation (*A*_n_) is largely dependent upon the value of *C*_c_ because the latter directly determines the affinity of Rubisco to CO_2_ or O_2_ (Farquhar et al., 1980; von Caemmerer, 2000). An increased *C*_c_ increases the rate of RuBP carboxylation and, thus, results in a rise in the photosynthetic rate. As shown by the calculation of *C*_c_ = *C*_i_ − *A*_n_/*g*_m_, *C*_c_ is mainly determined by three factors: intercellular CO_2_ concentration (*C*_i_), *A*_n_, and *g*_m_. During the steady-state phase under high light, *A*_n_ and *g*_m_ reach the steady-state values, the value of *C*_i_ determines *C*_c_ principally but is mainly influenced by *g*_s_. Stomata are the channels for gas exchange between leaf and atmosphere. During periods of drought or high temperatures, a decrease in *g*_s_ leads to an inhibition of the Calvin-Benson cycle (Flexas et al., 2002; Flexas and Medrano, 2002). In herbaceous plants of high values of *g*_m_, high rates of photosynthesis are usually accompanied by high values of *g*_s_ (Yamori et al., 2010, 2011). However, it is unclear whether the high-photosynthesis sclerophyllous plant *E. camaldulensis* elevate *g*_s_ to remedy the deficiency of *g*_m_.

According to the C_3_ photosynthesis model, photosynthesis can be limited by RuBP carboxylation and/or RuBP regeneration (Farquhar et al., 1980). When *C*_c_ is higher than *C*_trans_ (the chloroplast CO_2_ concentration at which the transition from RuBP carboxylation limitation to RuBP regeneration limitation occurs), then CO_2_ assimilation is limited by RuBP regeneration (Yamori et al., 2010, 2011). Once *C*_c_ is lower than *C*_trans_, CO_2_ assimilation tends to be limited by RuBP carboxylation. In herbaceous *N. tabacum* plants grown at high nitrogen concentration, the rate-limiting step of CO_2_ assimilation is RuBP regeneration because *C*_c_ is greater than *C*_trans_ (Yamori et al., 2010, 2011). In the sclerophyllous *E. camaldulensis*, the high rate of photosynthesis and low *g*_m_ can cause *C*_c_ to be less than *C*_trans_. Consequently, the rate of CO_2_ assimilation is probably limited by RuBP carboxylation in *E. camaldulensis*. If this occurs, then the reduced *C*_c_ drives increased photorespiration (or RuBP oxygenation). The photorespiratory pathway is essential for photosynthesis at normal atmospheric CO_2_ concentrations (Chastain and Ogren, 1989; Eisenhut et al., 2007), and impairment of that pathway decreases the rate of photosynthesis under such CO_2_ conditions (Somerville and Ogren, 1980, 1981, 1983; Takahashi et al., 2007).

Enhancement of the photorespiratory pathway leads to a considerably improved net photosynthetic rate in *Arabidopsis thaliana* (Timm et al., 2012, 2015). Therefore, we might also speculate that *E. camaldulensis* enhances the capacity of that pathway to favor the Calvin-Benson cycle. Furthermore, if the photorespiratory pathway is up-regulated in plants of *E. camaldulensis*, then the stoichiometry of the ATP/NADPH energy demand by primary metabolism will increase. Therefore, such plants must utilize other flexible mechanisms to balance the ATP/NADPH ratio, e.g., cyclic electron flow (CEF) around photosystem I (PSI) or the water-water cycle (Makino et al., 2002; Walker et al., 2014). The WWC channels electrons obtained from splitting of water molecules at PSII. These electrons are transported to oxygen via the Cyt *b*_6_/*f* complex and PSI, resulting in the formation of a proton gradient across the thylakoid membranes (Asada, 1999, 2000). However, little is known about how the WWC functions in the high-photosynthesis sclerophyllous plant *E. camaldulensis*.

*N. tabacum* is regarded as a model plant to study the mechanisms of photosynthetic regulation for herbaceous plants. However, it is not known how the sclerophyllous plant *E. camaldulensis* obtain a high rate of photosynthesis. Here, we compared *g*_s_, *g*_m_, CO_2_ assimilation, photorespiration, and alternative electron flow between *N. tabacum* and *E. camaldulensis*. Our objective was to examine the potential differences in mechanisms underlying high rates of photosynthesis between herbaceous and sclerophyllous plants.

## Materials and methods

### Plant materials and growth conditions

**Figure 1:**
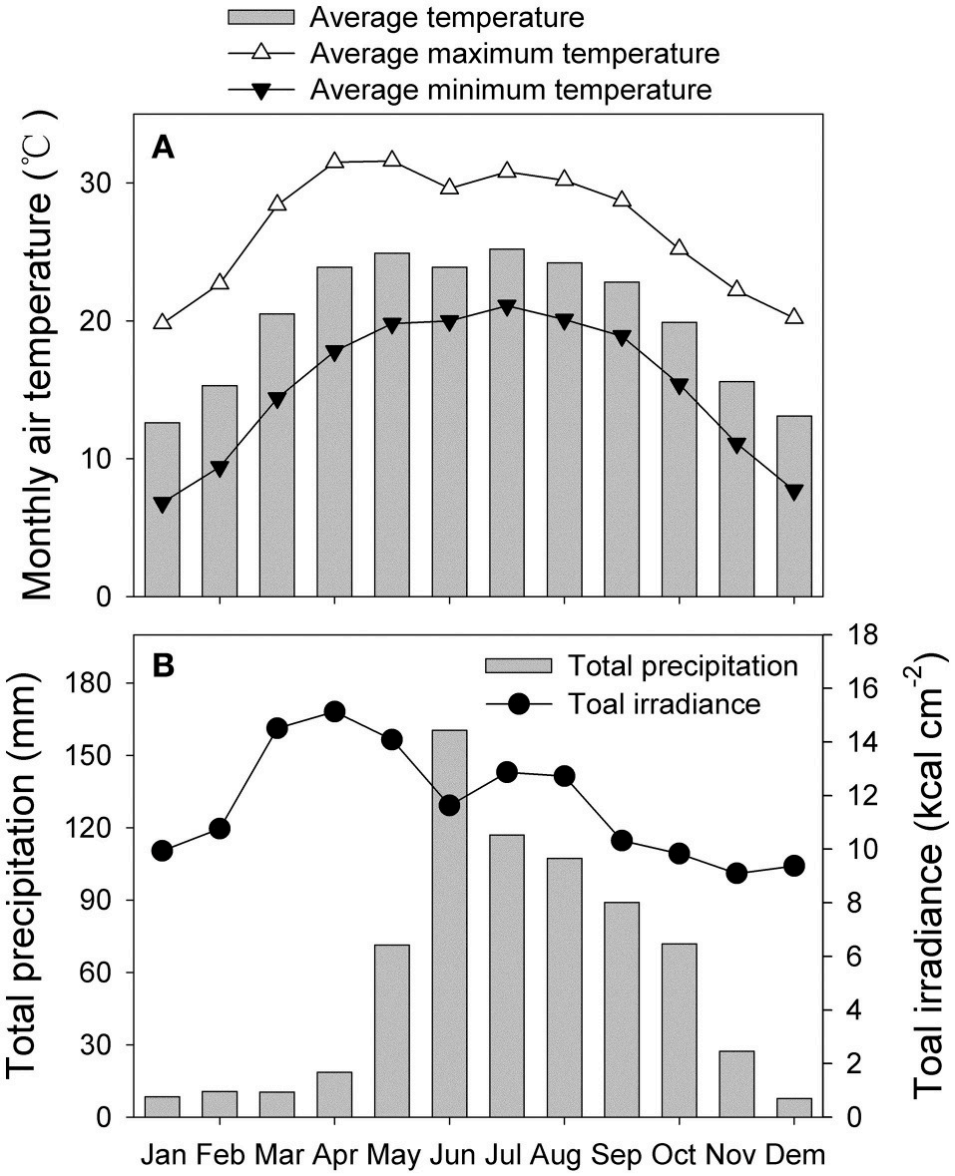
**Monthly climatic data collected from 1961 to 1980. (A)** monthly average air temperature, monthly average maximum and minimum temperatures; **(B)** monthly precipitation and total irradiance.

We compared the photosynthetic characteristics of *N. tabacum* and *Eucalyptus camaldulensis* Dehnh. The latter is a fast-growing species native to Australia that has been widely introduced into China for forest plantations. For this study, *Eucalyptus* samples were collected from plants grown in an open field at an elevation of 700 m in Dongchuan County, Kunming City, Yunnan Province, China. The monthly air temperature, total radiation and precipitation were displayed in Figure 1 (data were collected from 1961 to 1980). Seedlings of *N. tabacum* cv. k326 were cultivated in plastic pots in a phytotron at Kunming Institute of Botany, Yunnan, China. Growing conditions were 24/18°C (day/night), 60% relative humidity, and an atmospheric CO_2_ concentration maintained at 400 μmol mol^−1^. The phytotron used sunlight as the source of illumination, and plants were exposed to approximately 95% of full sunlight (maximum at noon ≈ 1990 μmol photons m^−2^ s^−1^). Photosynthetic parameters were measured in June of 2014. Measurements were made using four mature leaves from four independent plants per species. Fully expanded mature leaves on 13-week-old plants of *N. tabacum* were used for photosynthetic measurements. For *E. camaldulensis*, mature leaves that flushed in the spring on 3-year-old plants were used for measurements.

### Measurements of gas exchange and chlorophyll fluorescence

Photosynthetic parameters for gas exchange and chlorophyll fluorescence were monitored with an open gas exchange system that incorporated infrared CO_2_ and water vapor analyzers (Li-6400XT; Li-Cor Biosciences, Lincoln, NE, USA) and a 2-cm^2^ measuring head (6400-40 Leaf Chamber Fluorometer; Li-Cor Biosciences). Data were recorded at 25°C and a relative air humidity of 60–70%. The atmospheric CO_2_ concentration was maintained at 400 μmol mol^−1^ by the Li-6400XT. Both *g*_s_ and the CO_2_ assimilation rate peaked after plants were exposed to saturating light (2000 μmol photons m^−2^ s^−1^) for 20 min. Immediately, light response curves were evaluated at 2-min intervals at different light intensities. For *N. tabacum*, light response curves were measured at a photosynthetic photon flux density (PPFD) of 2000, 1600, 1200, 1000, 800, 600, 400, 300, 200, 150, 100, 50, or 0 μmol photons m^−2^ s^−1^. For *E. camaldulensis*, light response curves were measured at a photosynthetic photon flux density (PPFD) of 2000, 1600, 1200, 1000, 800, 500, 300, 150, 100, 50, or 0 μmol photons m^−2^ s^−1^.

The fluorescence parameters *F_m_′* and *F*_*s*_ were evaluated as previously described (Baker and Rosenqvist, 2004), with *F_m_′* representing the maximum fluorescence after light-adaption and *F*_*s*_ being the light-adapted steady-state fluorescence. The effective quantum yield of PSII was calculated as Φ_*PSII*_ = (*F_m_′* − *F*_*s*_)/*F_m_′* (Genty et al., 1989). The maximum fluorescence after dark adaptation (*F*_*m*_) was examined after 30 min of dark adaptation following measurement of the light response curve. Non-photochemical quenching was calculated as NPQ = (*F*_*m*_ − *F_m_′*)/*F_m_′*.

### Estimation of photosynthetic electron flow

Using the data of chlorophyll fluorescence parameters, total photosynthetic electron flow through PSII is calculated as follows (Krall and Edwards, 1992):

$$\begin{array}{l} J_{T}=\Phi_{PSII}\times PPFD\times L_{abs}\times 0.5 \end{array}$$

where Φ_PSII_ is the effective quantum yield of PSII and *L*_*abs*_ represents leaf absorbance. We applied the constant of 0.5 based on the assumption that photons were equally distributed between PSI and PSII.

Using the basic equation of leaf carbon dioxide gas exchange and Rubisco specificity for carboxylation relative to oxygenation (von Caemmerer and Farquhar, 1981; Sharkey, 1988; Walker et al., 2016), the rate of Rubisco carboxylation (*V*_c_) and that of Rubisco oxygenation (*V*_o_) are calculated according to.

$$\begin{array}{l} V_{c}=\frac{A_{\text{n}}+\;R_{\text{d}}}{1-(\Gamma^{*}/C_{\text{c}})}\\ V_{o}=\frac{A_{\text{n}}+\;R_{\text{d}}}{(C_{\text{c}}/2\Gamma^{*})-0.5} \end{array}$$

where *A*_n_ represented the net rate of CO_2_ assimilation, *R*_*d*_ was the rate of mitochondrial respiration as measured after 30 min of dark adaptation, Γ^*^ was the CO_2_ compensation point in the absence of daytime respiration (Farquhar et al., 1980; Brooks and Farquhar, 1985), and *C*_c_ was the chloroplast CO_2_ concentration. The electron flow for photorespiratory carbon oxidation can be expressed as:

$$\begin{array}{l} J\text{e}\left(\text{PCO}\right)=4\times V_{o} \end{array}$$

The NADPH demands from CO_2_ assimilation and photorespiration were calculated according to the models of Farquhar et al. (1980). Using the data from gas exchange measurements, we determined the rate of electron transport for NADPH required by carboxylation and oxygenation of RuBP (*J*_g_) as follows (Zivcak et al., 2013; Walker et al., 2014)

$$\begin{array}{l} J_{\text{g}}=4\times \left(A_{\text{n}}+\;R_{\text{d}}\right)\times \left(C_{\text{i}}+{2\Gamma }^{*}\right)/\left(C_{\text{i}}{-\Gamma }^{*}\right) \end{array}$$

where *C*_i_ was the intercellular CO_2_ concentration. The alternative electron flow was calculated as follows (Makino et al., 2002; Zivcak et al., 2013; Huang et al., 2016):

$$\begin{array}{l} \;J_{\text{a}}=J_{\text{T}}-J_{\text{g}} \end{array}$$

### Estimations of mesophyll conductance and chloroplast CO_2_ concentration

Values for mesophyll conductance (*g*_m_) were estimated through a combination analysis of gas exchange and chlorophyll fluorescence, and according to the following equation (Harley et al., 1992; Loreto et al., 1992; Warren and Dreyer, 2006; Yamori et al., 2010, 2011):

$$\begin{array}{l} {\text{g}}_{\text{m}}=\frac{A_{\text{n}}}{C_{\text{i}}-\Gamma^{*}\left(J_{\text{T}}+8\left(A_{\text{n}}+R_{\text{d}}\right)\right)/\left(J_{\text{T}}-4\left(A_{\text{n}}+R_{\text{d}}\right)\right)} \end{array}$$

Using the estimated *g*_m_, we calculated the chloroplast CO_2_ concentration (*C*_c_) according to the following equation (Long and Bernacchi, 2003; Warren and Dreyer, 2006; Yamori et al., 2010, 2011):

$$\begin{array}{l} C_{\text{c}}=C_{\text{i}}-\;\frac{A_{\text{n}}}{{\text{g}}_{\text{m}}} \end{array}$$

The response of net CO_2_ assimilation rate to CO_2_ concentration was examined at 2000 μmol photons m^−2^ s^−1^ and 25°C. Before *A*/*C*_i_ measurement, leaves were light adapted at 2000 μmol photons m^−2^ s^−1^ and 400 μmol mol^−1^ CO_2_ concentration for at least 20 min to obtain the maximum values of *g*_s_ and *A*_n_. Afterwards, the CO_2_ concentrations were set to 50 μmol mol^−1^ and increased stepwise. For *E. camaldulensis*, CO_2_ concentrations were set to 0, 50, 100, 150, 200, 300, 400, 600, 800, 1000, and 1200 μmol mol^−1^. The CO_2_ concentrations in *A*/*C*_i_ measurement in *N. tabacum* were set to 0, 50, 100, 150, 200, 300, 400, 600, 800, 1000, 1200, 1600, 2000 μmol mol^−1^. Each stepwise measurement was completed within 2–3 min. Using *A*/*C*_i_ curves, we calculated the maximum rates of RuBP regeneration (*J*_max_) and RuBP carboxylation (*V*_cmax_) according to the method of Long and Bernacchi (2003). To identify the limiting step of CO_2_ assimilation, we determined the chloroplast CO_2_ concentration at which the transition from RuBP carboxylation to RuBP regeneration occurred (*C*_trans_) as follows (Yamori et al., 2010, 2011):

$$\begin{array}{l} C_{\text{trans}}=\;\frac{K_{\text{c}}\left(1+O/K_{\text{o}}\right)J_{\text{max}}/4V_{\text{cmax}}-{2\Gamma }^{*}}{1-J_{\text{max}}/4V_{\text{cmax}}} \end{array}$$

where *K*_c_ (μmol mol^−1^) and *K*_o_ (mmol mol^−1^) were the Michaelis constants for CO_2_ and O_2_, respectively (Farquhar et al., 1980), and were assumed to be 406.8 μmol mol^−1^ and 277 mmol mol^−1^, respectively, at 25°C (Long and Bernacchi, 2003); *O* was the partial pressure of O_2_ and was assumed to be 210 (Farquhar et al., 1980); *J*_max_ was the maximum rate of RuBP regeneration; and *V*_cmax_ was the maximum rate of RuBP carboxylation. The rate-limiting step for CO_2_ assimilation was then determined by comparing the values of *C*_c_ and *C*_trans_.

### Modeling ATP supplied via flexible mechanisms

The total amount of ATP demand from Rubisco carboxylation and oxygenation was obtained with the following formula (Walker et al., 2014):

$$\begin{array}{l} v_{\text{ATP}}=\frac{\left(A_{\text{n}}\;+\;R_{\text{d}}\right)\;\left(3C_{\text{i}}\;+\;3.5\frac{\Gamma^{*}}{\alpha }\right)}{\left(C_{\text{i}}-\Gamma^{*}\right)} \end{array}$$

Assuming that the stoichiometry of ATP/NADPH produced by LEF (electron transport from PSII to NADP^+^) is 1.29 (Sacksteder et al., 2000; Seelert et al., 2000; Walker et al., 2014), the amount of ATP produced by LEF was calculated as:

$$\begin{array}{l} \;v_{\text{ATP}\left(\text{LEF}\right)}=1/2\times J_{\text{g}}\times 1.29 \end{array}$$

Rates of ATP supply from other flexible mechanisms were determined by subtracting the amount of ATP produced by LEF from *v*_*ATP*_ according to:

$$\begin{array}{l} \;v_{\text{ATP}\left(\text{Flex}\right)}=v_{\text{ATP}}-v_{\text{ATP}\left(\text{LEF}\right)} \end{array}$$

### Quantitative limitation analysis of *A*_n_

Photosynthetic limitations in *E. camaldulensis* and *N. tabacum* were assessed according to the method of Grassi and Magnani (2005) and Carriqui et al. (2015). The values for stomatal (*l*_*s*_), mesophyll conductance (*l*_mc_), and biochemical (*l*_b_) limitations represented measures of the relative importance of stomatal diffusion, mesophyll diffusion, and photosynthetic biochemistry in setting the observed value of *A*_n_. Relative photosynthetic limitations were calculated as follows (Grassi and Magnani, 2005; Carriqui et al., 2015):

$$\begin{array}{lll} l_{s} & = & \frac{{\text{g}}_{\text{tot}}/{\text{g}}_{\text{s}}\times \partial A_{\text{n}}/\partial C_{\text{c}}}{{\text{g}}_{\text{tot}}+\partial A_{\text{n}}/\partial C_{\text{c}}}\\ l_{mc} & = & \frac{{\text{g}}_{\text{tot}}/{\text{g}}_{\text{m}}\times \partial A_{\text{n}}/\partial C_{\text{c}}}{{\text{g}}_{\text{tot}}+\partial A_{\text{n}}/\partial C_{\text{c}}}\\ l_{b} & = & \frac{{\text{g}}_{\text{tot}}}{{\text{g}}_{\text{tot}}+\partial A_{\text{n}}/\partial C_{\text{c}}} \end{array}$$

where *g*_tot_ was total conductance to CO_2_ between the leaf surface and carboxylation sites (calculated as 1/*g*_tot_ = 1/ *g*_s_ + 1/ *g*_m_).

### Statistical analysis

All results were displayed as mean values of four independent measurements. We used one-way ANOVA and SPSS 16.0 software (SPSS Inc., Chicago, IL, USA) to examine differences between the two species. Those differences were considered significant at *P* < 0.05.

## Results

### *A*/*C*_i_ curves and the rate-limiting step of CO_2_ assimilation

**Figure 2:**
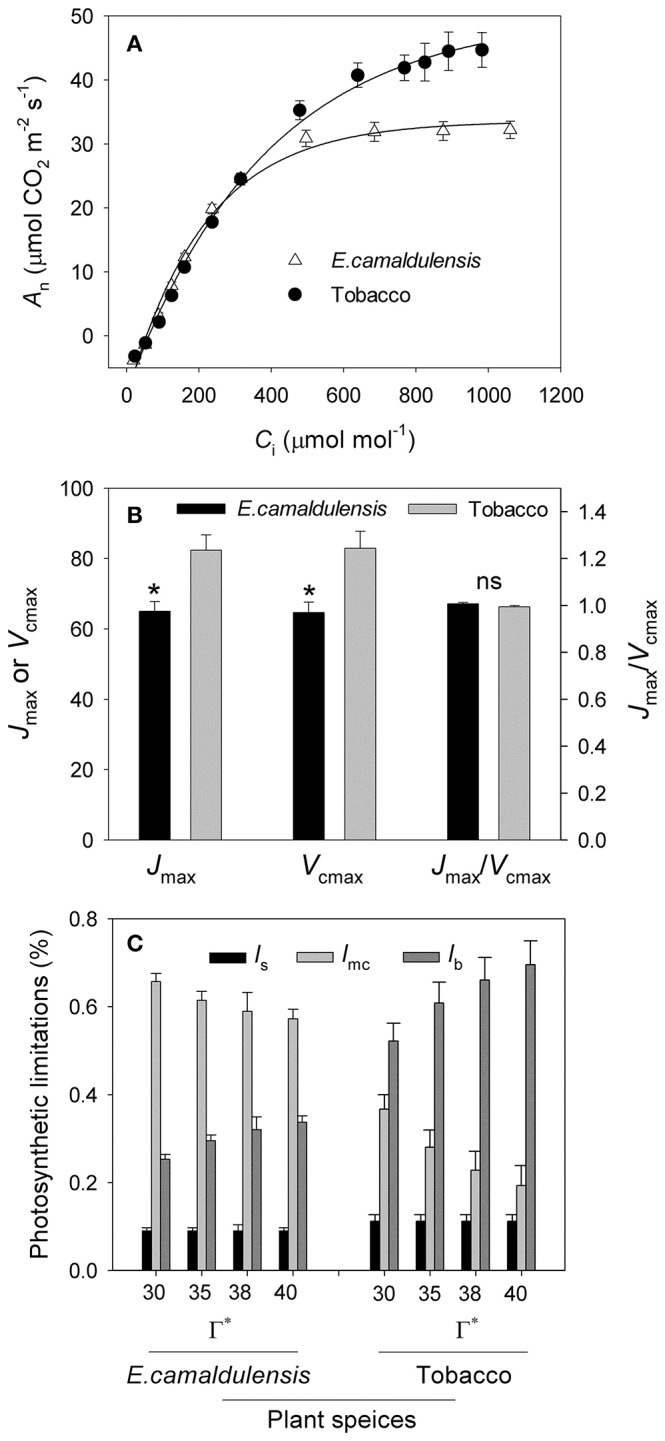
**Analysis of ***A***/***C***_**i**_ curves and quantitative limitation analysis of ***A***_**n**_ in ***Eucalyptus camaldulensis*** and ***N. tabacum***. (A)** Intercellular CO_2_ concentration (*C*_i_) response of CO_2_ assimilation rate (*A*_n_) in *Eucalyptus camaldulensis* and *N. tabacum* measured at 25°C and 1500 μmol photons m^−2^ s^−1^. **(B)** Values for *J*_max_, *V*_cmax_ and *J*_max_/*V*_cmax_ ratio. *J*_max_ represents the maximum rate of RuBP regeneration; *V*_cmax_ indicates the maximum rate of RuBP carboxylation. **(C)** Sensitivity analyses of relative stomatal (*l*_*s*_), mesophyll conductance (*l*_mc_) and biochemical (*l*_b_) limitations for photosynthesis to CO_2_ compensation point under the absence of respiration condition (Γ^*^) at 25°C and 400 μmol mol^−1^ CO_2_. Values are means ± SE (*n* = 4).

The *A*/*C*_i_ curves indicated that the maximum rate of photosynthesis was higher in *N. tabacum* than in *E. camaldulensis* (Figure 2A). Values for the maximum rate of RuBP regeneration (*J*_max_) and RuBP carboxylation (*V*_cmax_) were significantly higher in *N. tabacum* (Figure 2B). Both species showed similar ratios of *J*_max_/*V*_cmax_ (Figure 1B) as well as the same value for *A*_400_ at 400 μmol mol^−1^ CO_2_. Because the CO_2_ compensation point in the absence of daytime respiration (Γ^*^) has an important impact on *g*_m_, *C*_c_, *C*_trans_, and limitations on the stomata (*l*_*s*_), mesophyll conductance (*l*_mc_), and biochemical functions (*l*_b_), we conducted a sensitivity analysis and examined the rate-limiting step for CO_2_ assimilation to Γ^*^ (range from 30 to 40 μmol mol^−1^). For *E. camaldulensis, l*_mc_ was the most important constraining factor for photosynthesis, followed by *l*_*s*_ and *l*_b_ (Figure 2C). By contrast, biochemical limitations were the most significant in *N. tabacum*, followed by *l*_mc_ and *l*_*s*_ (Figure 2C). Furthermore, irrespective of Γ^*^, the value of *C*_c_ at 400 μmol mol^−1^ CO_2_ was significantly lower than *C*_trans_ in *E. camaldulensis* (Table 1), suggesting that the rate-limiting step of *A*_400_ tended to be RuBP carboxylation. By comparison, in *N. tabacum* the value of *C*_c_ at 400 μmol mol^−1^ CO_2_ was significantly higher than *C*_trans_ (Table 1), indicating *A*_400_ tended to be limited by RuBP regeneration.

**Table 1:** Sensitivity analyses of rate-limiting step for CO_**2**_ assimilation to Γ^*^.

**Species**	Γ^*^	***g*_m_**	***C*_c_**	***C*_trans_**	**Significance between *C*_c_ and *C*_trans_**	***A*_*r*_**	***A*_c_**
*E.camaldulensis*	30	0.107	86.2	160.2	0.0001	−	+
	35	0.115	102.2	146.9	0.0001	−	+
	38	0.121	111.1	138.8	0.0001	−	+
	40	0.124	116.0	133.5	0.045	−	+
*N. tabacum*	30	0.194	185.1	156.6	0.01	+	−
	35	0.263	215.9	143.3	0.0001	+	−
	38	0.338	234.4	135.3	0.0001	+	−
	40	0.424	246.8	130.0	0.0001	+	−

Sensitivity analyses of photosynthetic parameters calculated from A/C_i_ curves to CO_2_ compensation point in the absence of respiration condition (Γ^*^) in Eucalyptus camaldulensis and N. tabacum. g_m_, mesophyll conductance; C_c_, chloroplast CO_2_ concentration; C_trans_, chloroplast CO_2_ concentration at which the transition from RuBP carboxylation to RuBP regeneration occurs; A_r_, RuBP regeneration; A_c_, RuBP carboxylation. C_c_ for A_400_ less than C_trans_ indicates that CO_2_ assimilation is limited by A_c_, whereas C_c_ for A_400_ higher than C_trans_ indicates that CO_2_ assimilation is limited by A_r_. “−” represents no and “+” represents yes. Values are means ± SE (n = 4).

### Light response changes in CO_2_ assimilation and photosynthetic electron flow

**Figure 3:**
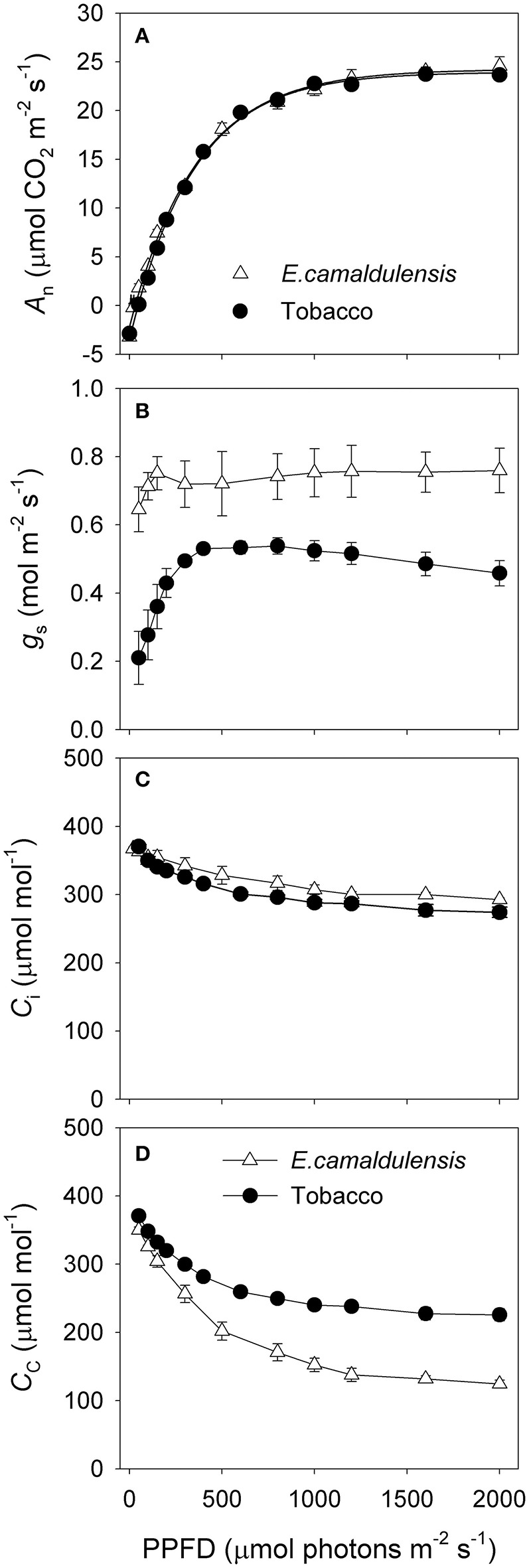
**Light response changes in (A)** photosynthetic rate (*A*_n_), **(B)** stomatal conductance (*g*_s_), **(C)** intercellular CO_2_ concentration (*C*_i_), and **(D)** chloroplast CO_2_ concentration (*C*_c_) for leaves of *Eucalyptus camaldulensis* and *N. tabacum*. Measurements were conducted at 25°C and 400 μmol mol^−1^ CO_2_. The value of *C*_c_ was based on the calculation of *g*_m_ on assumptions of Γ^*^ being 40 μmol mol^−1^ and *L*_*abs*_ being 0.85. Values are means ± SE (*n* = 4).

The light response curves demonstrated that the response of *A*_n_ to incident light was similar between *E. camaldulensis* and *N. tabacum* (Figure 3A). However, the maximum value of *g*_s_ was much higher in *E. camaldulensis* (Figure 3B), while *C*_i_ was slightly higher in that species (Figure 3C). When Γ^*^ was assumed to be 40 μmol mol^−1^, then *C*_c_ under saturating light was much lower in *E. camaldulensis* (Figure 3D). At 2000 μmol photons m^−2^ s^−1^ in light response curves, the value of *C*_c_ was 124 μmol mol^−1^ in *E. camaldulensis* and 226 μmol mol^−1^ in *N. tabacum* (Figure 3D). This large difference of *C*_c_ between *E. camaldulensis* and *N. tabacum* was principally caused by the contrast in *g*_m_.

**Figure 4:**
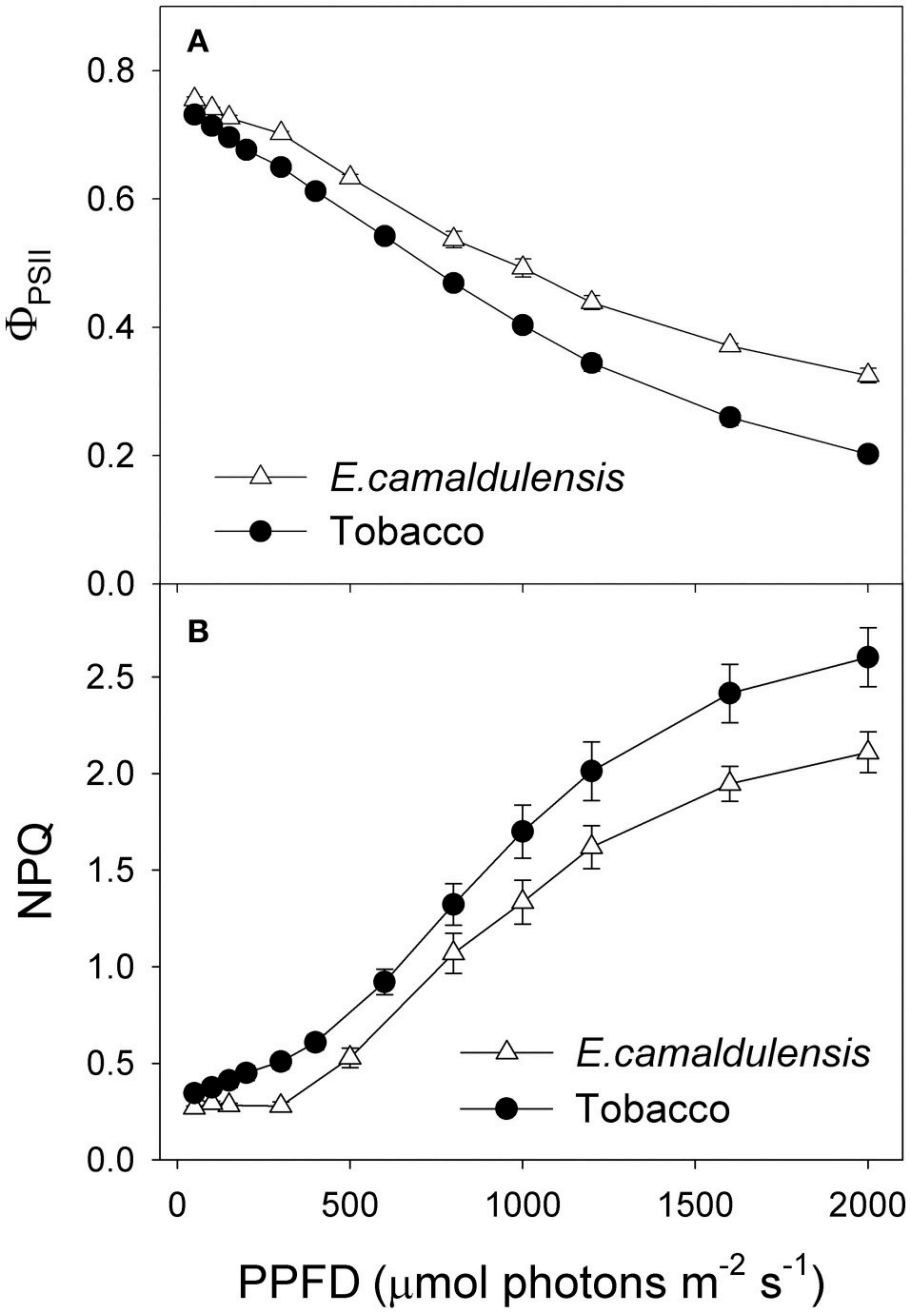
**Light response changes in (A)** effective quantum yield of PSII [Y(II)], and **(B)** non-photochemical quenching (NPQ) for leaves of *Eucalyptus camaldulensis* and *N. tabacum*. Measurements were conducted at 25°C and 400 μmol mol^−1^ CO_2_. Values are means ± SE (*n* = 4).

**Figure 5:**
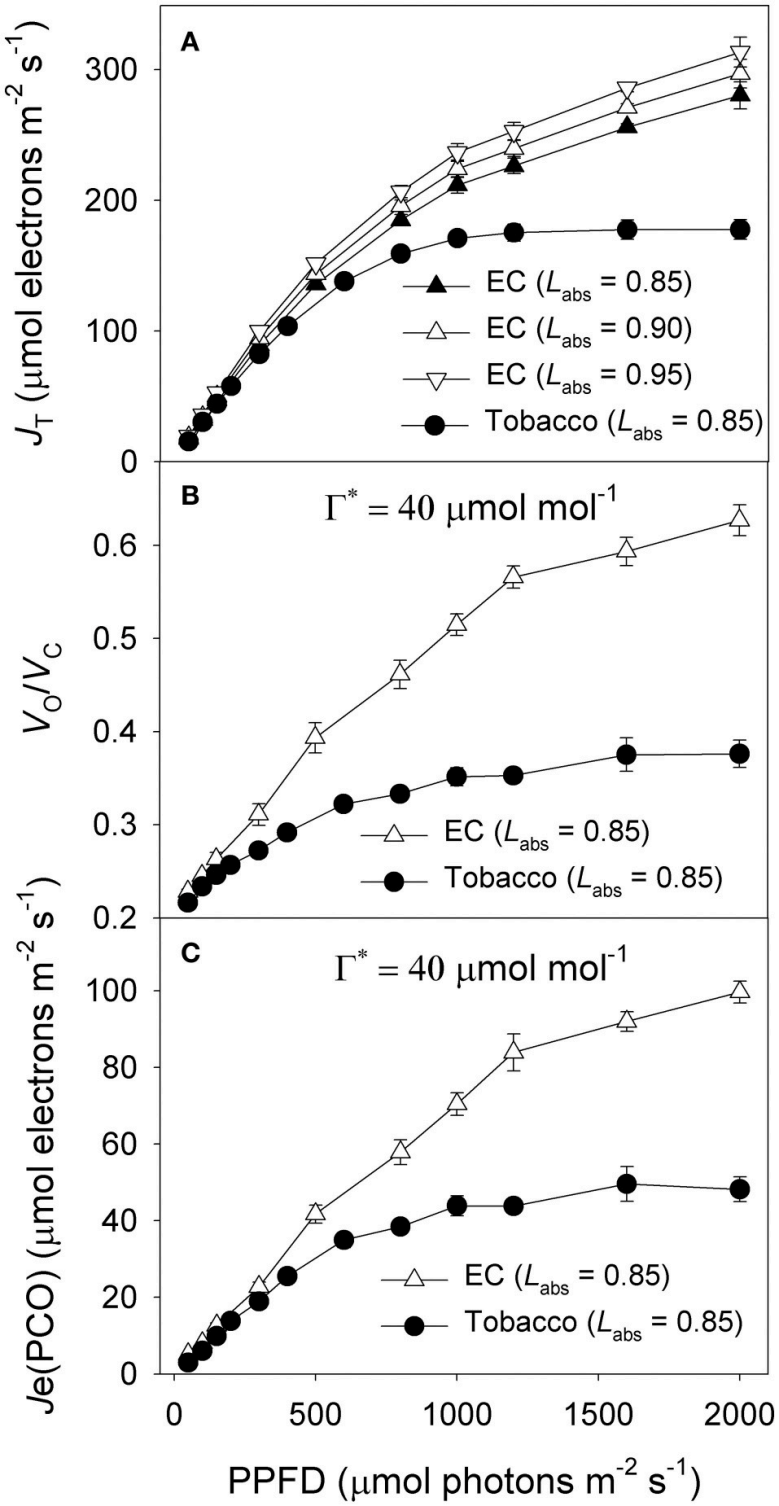
**Light response changes in (A)** photosynthetic electron flow through PSII (*J*_T_), and **(B)** the ratio of the rate of Rubisco carboxylation (*V*_c_) to that of Rubisco oxygenation (*V*_o_), and **(C)** electron flux for photorespiratory carbon oxidation [*J*e(PCO)] for leaves of *Eucalyptus camaldulensis* and *N. tabacum*. Measurements were conducted at 25°C and 400 μmol mol^−1^ CO_2_. Values are means ± SE (*n* = 4).

**Figure 6:**
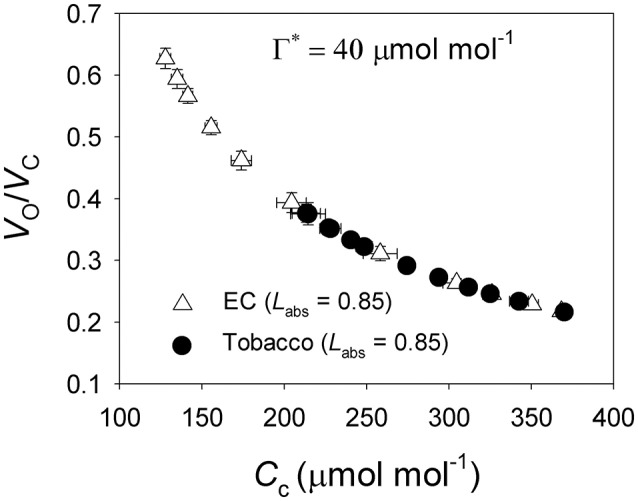
**The ratio of Rubisco oxygenation to carboxylation (***V***_**o**_/***V***_**c**_) as a function of ***C***_**c**_ for leaves of ***Eucalyptus camaldulensis*** and ***N. tabacum*****. The data of *V*_o_/*V*_c_ and *C*_c_ were referred from Figures 2D, 4B, respectively. Values are means ± SE (*n* = 4).

Under all light intensities, *E. camaldulensis* had significantly higher effective quantum yield of PSII (Φ_*PSII*_) compared to *N. tabacum*, especially under high light (Figure 4A). Concomitantly, NPQ values were lower in *E. camaldulensis* than *N. tabacum* (Figure 4B). According to the data of Φ_PSII_, *E. camaldulensis* had significantly higher values of total electron flow through PSII (*J*_T_) than *N. tabacum* (Figure 5A). Meanwhile, the ratios of the rate of Rubisco carboxylation (*V*_c_) to that of Rubisco oxygenation (*V*_o_) under saturating light intensities were much higher in *E. camaldulensis* (Figure 5B). On assumptions of Γ^*^ being 40 μmol mol^−1^ and leaf absorbance being 0.85 in *E. camaldulensis* and *N. tabacum*, at 2000 μmol photons m^−2^ s^−1^, comparative values for *J*_T_ and the *V*_o_/*V*_c_ ratio in *E. camaldulensis* vs. *N. tabacum* were 280 vs. 178 and 0.63 vs. 0.38, respectively (Figures 5A,B). Furthermore, under light intensities above 300 μmol photons m^−2^ s^−1^, electron flux for photorespiratory carbon oxidation [*J*e(PCO)] were significantly higher in *E. camaldulensis* (Figure 5C). Values for *J*e(PCO) at 2000 μmol photons m^−2^ s^−1^ were 100 and 48 μmol electrons m^−2^ s^−1^ in *E. camaldulensis* and *N. tabacum*, respectively. Plotting the *V*_o_/*V*_c_ ratio and *C*_c_ indicated that the difference in the *V*_o_/*V*_c_ ratio between *E. camaldulensis* and *N. tabacum* was mainly determined by the difference in *C*_c_ (Figure 6), which in turn caused by the change in *g*_m_.

### Alternative electron flow and ATP synthesis from flexible mechanisms

**Figure 7:**
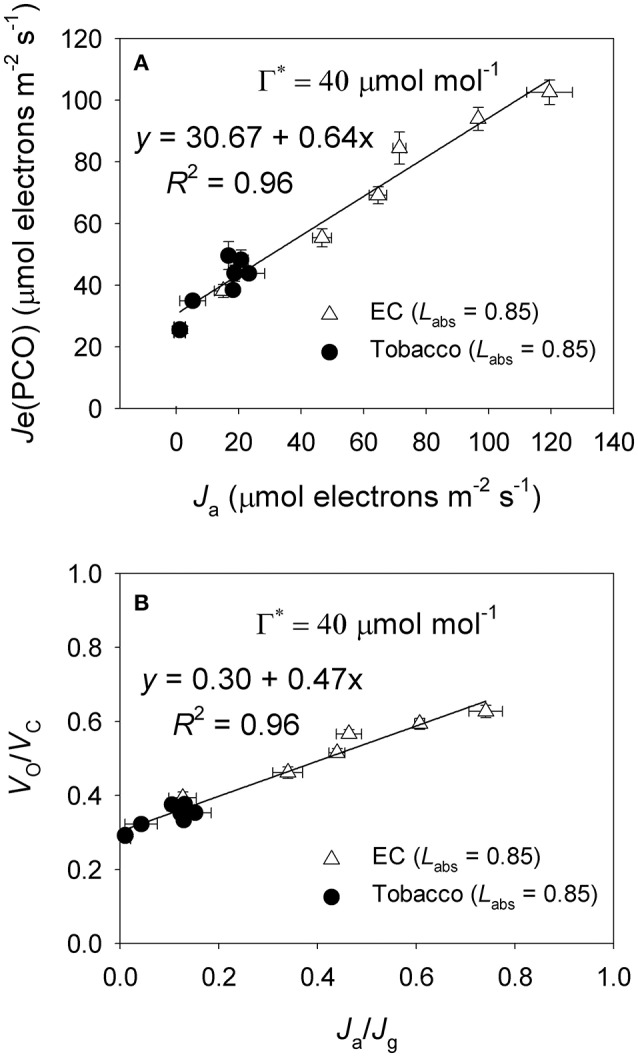
**(A)** The value of *J*e(PCO) as a function of *J*_a_ (electron flux for alternative electron sinks) for leaves of *Eucalyptus camaldulensis* and tobacco; **(B)** The value of *V*_o_/*V*_c_ as a function of *J*_a_/*J*_g_ for leaves of *Eucalyptus camaldulensis* and tobacco. Data were obtained from light response curves (light intensities higher than 300 μmol photons m^−2^ s^−1^) measured at 25°C and 400 μmol mol^−1^ CO_2_. Values are means ± SE (*n* = 4). The coefficient of correlation (*r*) and significance of correlation (*P*) are 0.98 and <0.0001 for Figure 6A, respectively. The coefficient of correlation (*r*) and significance of correlation (*P*) are 0.98 and <0.0001 for Figure 6B, respectively.

**Figure 8:**
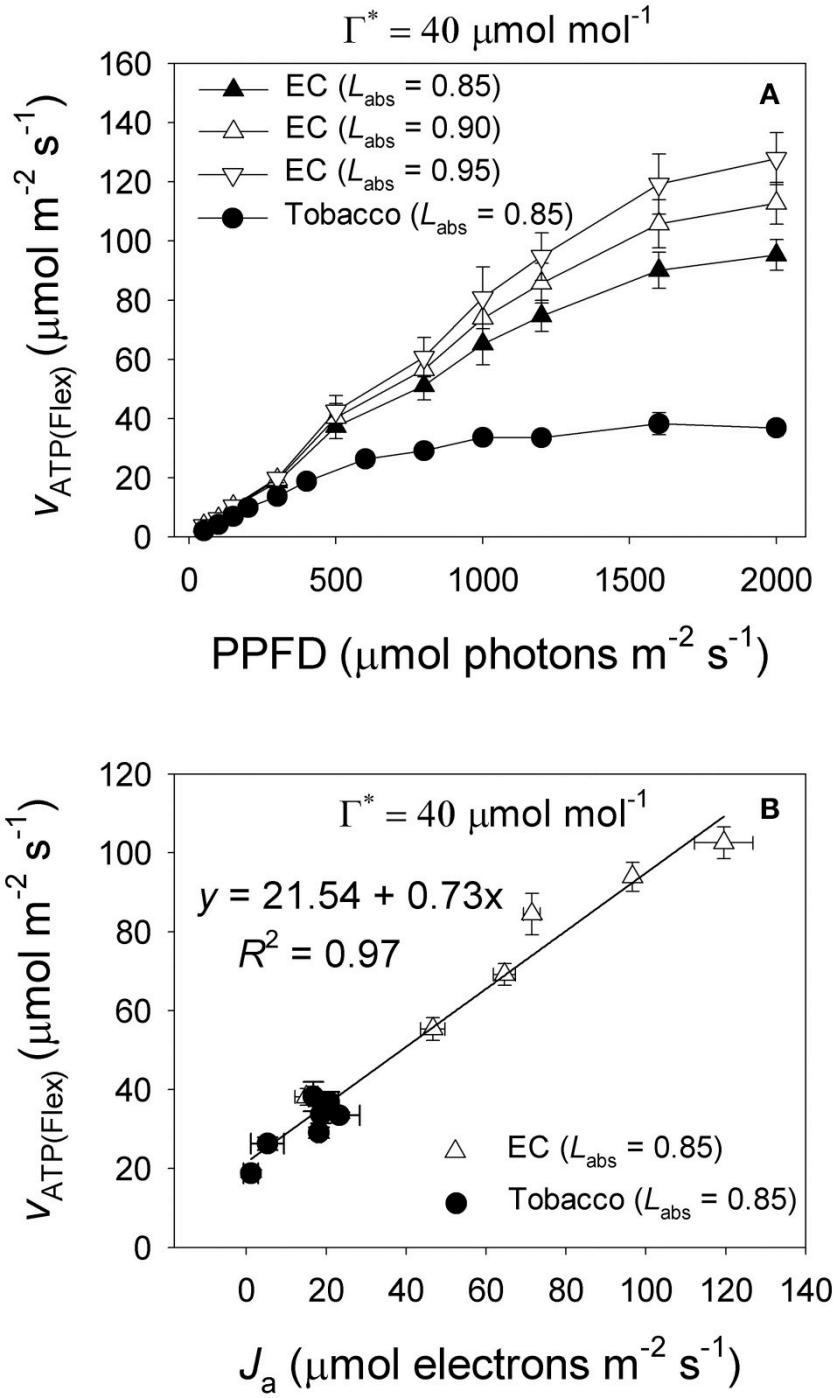
**(A)** Light response change in the rate of ATP supplied from other flexible mechanisms rather than electron flow from water to NADP^+^ [*v*_ATP(Flex)_] for leaves of *Eucalyptus camaldulensis* and tobacco; **(B)** Relationship between the electron flux for alternative electron sinks (*J*_a_) and *v*_ATP(Flex)_ for leaves of *Eucalyptus camaldulensis* and tobacco, and data were obtained from light response curves (light intensities higher than 300 μmol photons m^−2^ s^−1^) measured at 25°C and 400 μmol mol^−1^ CO_2_. Values are means ± SE (*n* = 4). The coefficient of correlation (*r*) and significance of correlation (*P*) are 0.98 and <0.0001 for Figure 7B, respectively.

Under high light, *E. camaldulensis* had significantly increased alternative electron flow, as indicated by the higher values of *J*_a_ and the *J*_a_/*J*_g_ ratio (Figure 7). To examine the relationship between the alternative electron flow and photorespiration, we evaluated possible associations between *J*_a_ and *J*e(PCO) under light intensities higher than 300 μmol photons m^−2^ s^−1^. Interestingly, *J*_a_ was positively and linearly correlated with *J*e(PCO) (Figure 7A, *P* < 0.0001). Similarly, the *J*_a_/*J*_g_ ratio was positively and linearly correlated with *V*_o_/*V*_c_ ratio (Figure 7B, *P* < 0.0001). According to photosynthesis model, an increase in the *V*_o_/*V*_c_ ratio requires a higher ATP/NADPH energy demand from other flexible mechanisms such as cyclic electron flow and alternative electron flow. Increased alternative electron flow promotes the formation of a proton gradient across the thylakoid membrane (ΔpH), which can be used for activating NPQ and ATP synthesis. We found that under high light the rate of ATP supplied from alternative electron sinks [*v*_ATP(Flex)_] were much higher in *E. camaldulensis* than in *N. tabacum* (Figure 8A), and the rate of alternative electron flow was positively correlated to *v*_ATP(Flex)_ (Figure 8B, *P* < 0.0001). Furthermore, NPQ values under high light were significantly lower in *E. camaldulensis* than in *N. tabacum* (Figure 4B), suggesting that the main role for alternative electron flow in *E. camaldulensis* is not to activate NPQ but to provide extra ATP. The greater capacity of the photorespiratory pathway in *E. camaldulensis* plants is sustained by the enhanced alternative electron flow.

## Discussion

In herbaceous C_3_ crop species such as *N. tabacum*, spinach, rice and wheat, high stomatal and mesophyll conductance are essential for their strong photosynthetic capacities (Yamori et al., 2010, 2011). However, for sclerophyllous *Eucalyptus camaldulensis*, we found that its high rate of CO_2_ assimilation (Figure 3A) was accompanied with a low *g*_m_ (≈0.12 molm^−2^ s^−1^) (Table 1). Thus, high levels of mesophyll conductance do not appear to be a common mechanism for high rates of photosynthesis in C_3_ plants. Surprisingly, in the sclerophyllous *E. camaldulensis*, the high rates of photosynthesis occurred at the low levels of *C*_c_ (Figure 3D), which directly increased the *V*_o_/*V*_c_ ratio (Figure 5B). What is more, *E. camaldulensis* showed increased capacities of photorespiratory pathway (Figure 5C) and electron flow to alternative sinks (Figure 7). Enhancement of photorespiratory pathway increased the release of CO_2_ in mitochondria. This photorespired CO_2_ can be trapped and reassimilated by chloroplast, thereby boosting photosynthesis (Busch et al., 2013). Concomitantly, the increased photorespiratory pathway in *E. camaldulensis* needs more extra ATP supply from alternative electron sinks rather than electron transfer from water to NADP^+^. Interestingly, the increased alternative electron flow provided essential extra ATP to balance the energy budget and sustain the high rate of photorespiratory pathway, then increasing the rate of photosynthetic CO_2_ assimilation. These results highlight that the sclerophyllous *E. camaldulensis* enhanced the capacities of photorespiratory pathway and alternative electron flow to sustain a high rate of photosynthesis.

### Quantitative limitation analysis of *A*_n_

In *N. tabacum* leaves, *l*_b_ was the most important constraining factor for photosynthesis, followed by *l*_mc_ and *l*_*s*_, being consistent with high levels of stomatal and mesophyll conductance. By comparison, in *E. camaldulensis, l*_mc_ had the greatest influence while *l*_*s*_ was the least limiting factor. This was consistent with the higher *g*_s_ and lower *g*_m_ measured for that species. These results confirmed that, the major limiting factor for photosynthesis differs intrinsically between high-photosynthesis herbaceous and sclerophyllous plants. Diffusional limitations of CO_2_ are the main reason for lower photosynthetic rates in ferns than in angiosperms (Gago et al., 2013; Carriqui et al., 2015). Mesophyll conductance is the most constraining factor for photosynthesis in ferns (Carriqui et al., 2015). These findings suggested that, not only in low-photosynthesis species (e.g., ferns) but also in some high-photosynthesis sclerophyllous species such as *E. camaldulensis*, mesophyll conductance is the primary limiting factor for photosynthesis. The difference of *g*_m_ between *E. camaldulensis* and *N. tabacum* is probably determined by their leaf anatomy (Terashima et al., 2006). The thicker cell walls lead to a lower *g*_m_ in *E. camaldulensis* when compared with *N. tabacum*.

### The rate-limiting step of CO_2_ assimilation

The rate of CO_2_ assimilation can be limited by RuBP carboxylation and/or RuBP regeneration in C_3_ plants (Farquhar et al., 1980; Yamori et al., 2010, 2011). The specific rate-limiting step of CO_2_ assimilation is determined by the relative values of *C*_c_ and *C*_trans_. When the value of *C*_c_ is higher than *C*_trans_, the CO_2_ assimilation rate is mainly limited by RuBP regeneration. Otherwise, the CO_2_ assimilation rate is limited by RuBP carboxylation when *C*_c_ is lower than *C*_trans_. In *N. tabacum* leaves, the value of *C*_c_ under saturating light was higher than *C*_trans_, and thus the rate-limiting step of CO_2_ assimilation tended to be RuBP regeneration (Table 1). On the contrary, the value of *C*_c_ under saturating light was lower than *C*_trans_ in *E. camaldulensis*, and thus the rate of CO_2_ assimilation was limited by RuBP carboxylation (Table 1). According to the calculation of *C*_c_ = *C*_i_ − *A*_n_/*g*_m_, the value of *C*_c_ can be affected by three parameters *C*_i_, *A*_n_, and *g*_m_. Here, because *A*_n_ and *C*_i_ values under saturating light were similar in *E. camaldulensis* and *N. tabacum* (Figures 3A,C), Because the value of *C*_c_ can be largely determined by *g*_m_, the difference in the main rate-limiting step of CO_2_ assimilation between *N. tabacum* and *E. camaldulensis* was mainly caused by the contrasts in their mesophyll conductance.

In the herbaceous *N. tabacum*, which has thin, flexible leaves, *g*_m_ can reach 0.5 mol m^−2^ s^−1^ when plants are exposed to high nitrogen concentrations (Yamori et al., 2011). By comparison, sclerophyllous plants have relatively lower *g*_m_ values that range between 0.09 and 0.25 mol m^−2^ s^−1^ (Lloyd et al., 1992; Hassiotou et al., 2009). Conductance in the mesophyll can be influenced by leaf anatomical traits such as the cell surface area and the chloroplast surface area that is exposed to intercellular air spaces (Evans et al., 1994; Oguchi et al., 2005; Terashima et al., 2011), chloroplast rearrangements (Tholen et al., 2008), cell wall thickness (Terashima et al., 2006, 2011; Flexas et al., 2012), and LMA (Flexas et al., 2008; Hassiotou et al., 2009). The value of LMA for in *E. camaldulensis* is approximately 169.5 g m^−2^ (Suganuma et al., 2006), which is much higher than *N. tabacum* (approximately 25 g m^−2^) (Yamori et al., 2010). The large differences of leaf anatomical traits between *E. camaldulensis* and *N. tabacum* might result in the diversity of *g*_m_.

### Photorespiration

We found that the capacity of the photorespiratory pathway was greater for *E. camaldulensis* than for *N. tabacum* (Figures 5B,C). Rubisco is a dual functional enzyme that catalyzes the carboxylation of RuBP, but also oxygenates RuBP in photorespiration. A lower *C*_c_ in *E. camaldulensis* would increase the rate of RuBP oxygenation by Rubisco. Photorespiration begins with RuBP oxygenation that generates glycolate-2-phosphate and glycerate-3-phosphateare. To maintain a steady-state high rate of photosynthesis in *E. camaldulensis*, the RuBP pool must remain stable. In chloroplast, the steady-state of RuBP pool depends on two different RuBP regeneration pathways: the Calvin-Benson cycle and the photorespiratory pathway. Impairment of photorespiratory pathway induced a gradient decrease in photosynthetic rate at atmospheric CO_2_ concentration (Takahashi et al., 2007). Importantly, during steady-state phases, the rates of RuBP oxygenation and RuBP regeneration through the photorespiratory pathway must be balanced. Under high light, *E. camaldulensis* accelerate the photorespiratory pathway to favor the regeneration of RuBP via glycerate-3-phosphate, thereby preventing the RuBP pool from shrinking.

A reduction in *C*_c_ also accelerates the production of photorespiratory intermediates, e.g., glycine and glycerate, which inhibit the Calvin-Benson cycle (Chastain and Ogren, 1989; Eisenhut et al., 2007; Timm et al., 2012, 2015). In *Arabidopsis thaliana* plants with elevated glycine decarboxylase activity, the rapid acceleration of the photorespiratory pathway lowers the accumulation of photorespiratory metabolites including those which impair Rubisco activation and possibly the activity of other enzymes of the Calvin-Benson cycle, increasing the performance of the Calvin-Benson cycle (Timm et al., 2012, 2015). Furthermore, *N. tabacum* plants grown under high light and high nitrogen concentration up-regulate photorespiratory pathway to maintain high rates of photosynthesis (Huang et al., 2014, 2016). To overcome those detrimental effects, the photorespiratory pathway should be enhanced in *E. camaldulensis*. In addition, although CO_2_ is released in the mitochondria in the photorespiratory pathway, C_3_ plants can trap photorespired CO_2_ within individual mesophyll cell. This causes chloroplast CO_2_ concentrations to rise and, ultimately, improves the rate of photosynthesis in C_3_ plants (Busch et al., 2013). Taking together, the enhanced photorespiratory capacity strongly contributes to the high rate of CO_2_ assimilation in *E. camaldulensis*.

### Alternative electron flow

We were surprised to learn that, under saturating illumination, alternative electron flow was enhanced in *E. camaldulensis* when compared with *N. tabacum*. The water-water cycle, nitrate reduction and malate shunt are potential candidates responsible for this increase in alternative electron flow (Yi et al., 2014). When plants are illuminated at atmospheric CO_2_ and O_2_, most of this alternative flow accounts for the electron flux to oxygen and oxidized ascorbic acid (Miyake and Yokota, 2000; Makino et al., 2002). Thus, we concluded that the WWC activity was greater in *E. camaldulensis*. During the early phase of photosynthetic induction in rice, the WWC first generates a ΔpH across the thylakoid membranes to form NPQ and supply ATP for carbon assimilation (Neubauer and Yamamoto, 1992; Miyake and Yokota, 2000; Makino et al., 2002). However, when photosynthesis reaches a steady-state rate, the WWC no longer maintains a high NPQ but instead provides additional ATP for primary metabolism in rice leaves (Makino et al., 2002). Our light response curves indicated that NPQ values under high light were significantly lower in *E. camaldulensis* (Figure 4B). Because the NPQ activation relies on the acidification of thylakoid lumen, and which also depresses electron transport through Cyt *b*_6_/*f* complex via “photosynthetic control,” this result suggested the higher levels of lumen acidification in leaves of *N. tabacum* when illuminated at high light. Therefore, the steady-state rate of WWC under intense illumination in *E. camaldulensis* mainly contributed to additional ATP synthesis rather than lumen acidification, which is consistent with previous studies on the role of the WWC (Makino et al., 2002; Huang et al., 2016).

In electron flow from PSII to NADP^+^, the stoichiometry of the ATP/NADPH ratio is thought to be 1.29 (Sacksteder et al., 2000; Seelert et al., 2000). By comparison, each Rubisco oxygenation consumes 3.5 ATP and 2 NADH equivalents in total. Therefore, the occurrence of photorespiratory pathway needs a higher ATP/NADPH ratio than 1.29 to maintain primary metabolism (Edwards and Walker, 1983; Walker et al., 2014). Under high light and ambient CO_2_, the ATP/NADPH ratio required by CO_2_ assimilation, photorespiration, and nitrite assimilation is approximately 1.6 (Walker et al., 2014). Furthermore, the ATP/NADPH energy demand for primary metabolism will rise as photorespiration increases. As a result, the rate of ATP supplied from other flexible pathways must be higher in *E. camaldulensis* due to its higher rate of photorespiratory pathway. Cyclic electron flow and the WWC are the main flexible pathways that contribute to extra ATP synthesis under high light and atmospheric CO_2_ concentrations (Makino et al., 2002; Walker et al., 2014; Huang et al., 2015, 2016). Here, we found that the greater rate of photorespiration was accompanied by higher alternative electron flow (Figure 7), and the rate of ATP supplied from other flexible mechanisms was positively correlated to the rate of electron flow to alternative sinks (Figure 8B). Therefore, it appears that *E. camaldulensis* enhances the alternative electron flow to balance the ATP/NADPH energy demand for high rates of photorespiration.

## Conclusions

In order to illustrate the potential different mechanisms underlying the high rates of photosynthesis in sclerophyllous and herbaceous C_3_ plants. Gas exchange and chlorophyll fluorescence were measured in *E. camaldulensis* (sclerophyllous) and *N. tabacum* (herbaceous). Although *E. camaldulensis* and *N. tabacum* had similar *A*_n_ under saturating light, the value of *g*_m_ differed largely between *N. tabacum* and *E. camaldulensis*. In *N. tabacum*, a higher *g*_m_ increased the value of *C*_c_, resulting in the rate-limiting step of CO_2_ assimilation tended to be RuBP regeneration. On the contrary, RuBP carboxylation was the main rate-limiting step of CO_2_ assimilation in *E. camaldulensis* because CO_2_ diffusion to the chloroplasts was restricted by a lower *g*_m_. Therefore, the rate-limiting step of CO_2_ assimilation appears to be more related to *g*_m_ rather than *g*_s_ in high-photosynthesis species. The lower *C*_c_ aggravated RuBP oxygenation in *E. camaldulensis*. Meanwhile, increased flux through the photorespiratory pathway minimizes the accumulation of photorespiratory metabolites, benefiting photosynthetic CO_2_ fixation in the Calvin-Benson cycle in *E. camaldulensis*. In order to balance the ATP/NADPH energy demand for high rates of photorespiration, *E. camaldulensis* up-regulated alternative electron flow to provide extra ATP. Thus, coordination of photorespiratory pathway and alternative electron flow is crucial for the high rates of CO_2_ assimilation in *E. camaldulensis*. These results highlight the different mechanisms responsible for high rates of photosynthesis in the sclerophyllous plant *E. camaldulensis* and the herbaceous plant *N. tabacum*.

## Author contributions

WH and YT conceived and designed research. WH conducted experiments. WH, GY, and WY analyzed data. WH wrote the manuscript.

### Conflict of interest statement

The authors declare that the research was conducted in the absence of any commercial or financial relationships that could be construed as a potential conflict of interest.
